# Novel RNA-Based Therapies in the Management of Dyslipidemias

**DOI:** 10.3390/ijms26031026

**Published:** 2025-01-25

**Authors:** Constantine E. Kosmas, Maria D. Bousvarou, Donatos Tsamoulis, Maria Gianniou, Evangelia J. Papakonstantinou, Loukianos S. Rallidis

**Affiliations:** 1 2nd Department of Cardiology, National & Kapodistrian University of Athens, 12462 Athens, Greece; donatostsamoulis@gmail.com (D.T.); lrallidis@gmail.com (L.S.R.); 2 Health Center of Sofades, 43300 Karditsa, Greece; marabous60@gmail.com; 3 School of Medicine, University of Patras, 26504 Rio, Greece; mgianniou45@gmail.com; 4 General Directorate of Public Health and Social Welfare, Attica Region, 11521 Athens, Greece; lpapakon777@gmail.com

**Keywords:** antisense oligonucleotides (ASOs), small interfering RNAs (siRNAs), dyslipidemia, lipoprotein(a) [Lp(a)], proprotein convertase subtilisin/kexin type 9 (PCSK9), apolipoprotein C-III (ApoC-III), angiopoietin-like protein 3 (ANGPTL3), cardiovascular risk

## Abstract

Pharmaceutical advancements and an improved understanding of pathophysiology have enabled innovative therapies for chronic conditions like dyslipidemia. This condition is marked by abnormalities in lipid homeostasis. Nucleic acid therapeutics, including antisense oligonucleotides and small interfering RNAs, are novel management strategies that silence genes by targeting mRNA. Antisense oligonucleotides modify mRNA to inhibit protein production, whereas small interfering RNAs induce mRNA degradation via the RNA-induced silencing complex (RISC), thus offering promising treatments for dyslipidemia and atherosclerotic cardiovascular disease. Chemical modifications improve their stability and mRNA targeting. RNA-based therapies targeting PCSK9, Lp(a), ApoC-III, and ANGPTL3 hold transformative potential for treating dyslipidemia effectively. This article discusses the latest data from completed and ongoing trials on RNA therapies for dyslipidemia, including inclisiran, pelacarsen, olpasiran, zerlasiran, lepodisiran, volanesorsen, olezarsen, plozasiran, zodasiran, and solbinsiran. Each therapy targets specific molecules while also significantly impacting other lipid parameters. The promising results of these trials indicate potential improvements in lipid therapy and cardiovascular risk reduction, with ongoing studies expected to further refine the role of the novel RNA-based agents in effective lipid management.

## 1. Introduction

The mindboggling speed of pharmaceutical advancements, combined with the ever-expanding knowledge of pathophysiological mechanisms, enables the transformation of innovative ideas into cutting-edge therapies, aiming to improve the management of challenging, chronic health conditions and, ultimately, elevate routine clinical practice.

Dyslipidemia is characterized by aberrant values of lipid levels, resulting from imbalances in lipid metabolism. This definition encompasses elevated concentrations of serum triglycerides (TGs) and/or total cholesterol (TC), marked by increased non-high-density lipoprotein cholesterol (non-HDL-C) levels and diminished serum high-density lipoprotein cholesterol (HDL-C), either in isolation or in tandem. Non-HDL-C stands as a valuable marker of atherogenicity, comprising low-density lipoprotein cholesterol (LDL-C)—the central player in atherogenesis—alongside very-low-density lipoprotein cholesterol (VLDL-C), intermediate-density lipoprotein cholesterol (IDL-C), and lipoprotein(a) [Lp(a)] [1]. Elevated serum levels of Lp(a), or hyperlipoproteinemia (a), represent a notable component of dyslipidemia due to Lp(a)’s high atherogenicity index, as there is substantial evidence [2,3,4] clearly indicating that Lp(a) is an independent atherogenic factor. Novel therapies aiming at reducing Lp(a) [5], including RNA-based treatments—which form the topic of the present review—are currently under investigation and will be discussed in detail later on in this review.

Dyslipidemia is a widespread health concern. According to the American Heart Association’s 2024 Heart Disease and Stroke Statistics Report (for the years 2017 to 2020), the prevalence rates in male and female adults, respectively, were similar for the elevated total cholesterol (TC ≥ 200 mg/dL: 32.8% in males, 36.2% in females) and LDL-C (LDL-C ≥ 130 mg/dL: 25.6% in males, 25.4% in females), whereas decreased HDL-C levels (<40 mg/dL) were more common in males (25%) than females (9.3%). Increased TG levels occurred in 19.9% of adults. Additionally, elevated Lp(a) affects over 20% of the population. Dyslipidemia is a well-established major precursor and risk factor for atherosclerotic cardiovascular disease (ASCVD), the leading cause of death worldwide. For 2021, the global data indicate an age-adjusted mortality rate of 237.9 per 100,000 individuals due to CVD, with elevated LDL-C levels contributing to an estimated 3.72 million deaths [6]. Taking into account the above, dyslipidemia constitutes a compelling subject for exploring novel management strategies.

Pharmacological agents with nucleic acid properties, or nucleic acid therapeutics (NATs) [7], have been widely explored in the search for pioneering dyslipidemia treatments. The focus has primarily shifted towards antisense oligonucleotides (ASOs) and small interfering RNAs (siRNAs). Their mechanisms of action are fundamentally based on the principle of complementarity and promote gene silencing. The complementarity rule implies that in nucleic acids, adenine always pairs with thymine in double-stranded DNA and with uracil in RNA, while guanine pairs with cytosine in both DNA and RNA.

**Figure 1:**
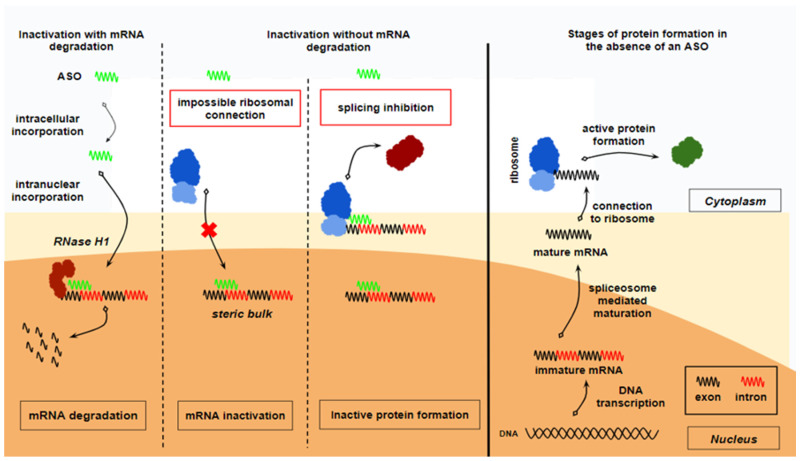
Mechanism of action of ASOs. Antisense oligonucleotides (ASOs) bind intranuclearly to the immature mRNA molecule and prevent the formation of an active protein. This occurs either via mRNA degradation or via the inactivation of the mRNA without degradation. Degradation of the mRNA is catalyzed by the enzyme RNase H1. Inactivation without degradation occurs by either forming a steric bulk that prevents ribosome attachment to the mature mRNA or by splicing inhibition, which prevents the mRNA maturation process, thereby leading to the production of an inactive protein. Normally, in the absence of antisense oligonucleotides (ASOs), the enzymatic transcription of a DNA gene produces an immature mRNA molecule. This immature mRNA undergoes maturation through the action of the spliceosome, which removes introns, resulting in a mature mRNA molecule. The mature mRNA is then transported to the cytoplasm, where it binds to a ribosome and is translated into a protein. This process ultimately leads to the formation of an active protein.

ASOs were among the first nucleic acid therapies (NATs) to be researched and approved for clinical use. These exogenous, single-stranded oligodeoxyribonucleotides are typically 18 to 30 bases in length. ASOs work by inhibiting the production of active specific proteins through the modification of their precursor mRNA molecule. Their mechanisms of action include binding complementarily to mRNA and, subsequently, are as follows: (a) inducing RNase-H-mediated degradation, (b) modulating mRNA splicing, (c) blocking translation through steric interference, and (d) stimulating an immune response via multiple cytosine–phosphodiester–guanine (CpG) sequences [7]. Depending on their mechanism, ASOs may act in the nucleus, cytoplasm, or both [8]. The mechanism of action of ASOs is shown in Figure 1.

**Figure 2:**
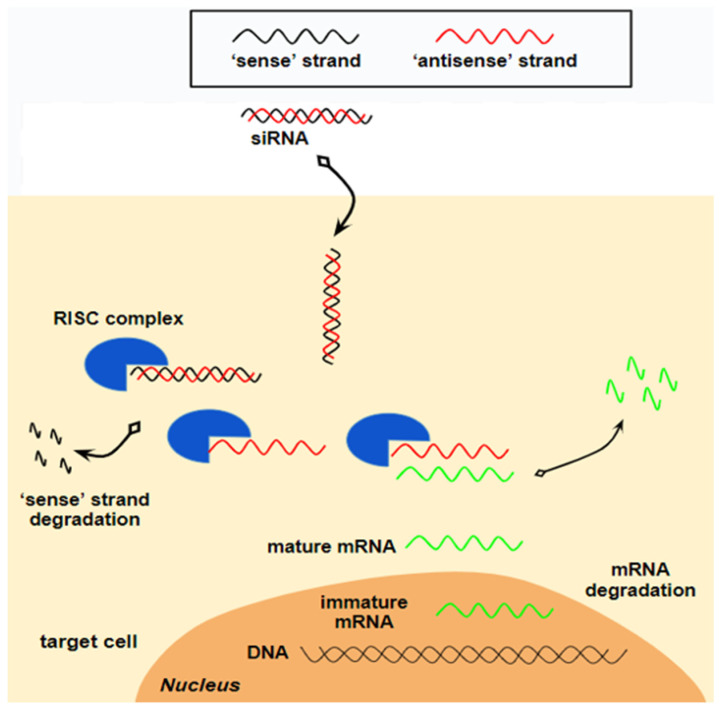
Mechanism of action of siRNAs. siRNAs consist of a double-stranded RNA molecule, containing a sense strand and an antisense strand. The antisense strand acts as a guide, facilitating the association of siRNA with a protein complex, called “the RISC complex” (RNA-induced silencing complex). Upon association with the RISC complex, the sense strand is degraded, while the antisense strand remains bound to the target mature mRNA. This binding leads to the degradation of the mRNA by the RISC complex, preventing it from being translated into a protein.

siRNAs are duplex RNA molecules consisting of a passenger (sense) strand and a guide (antisense) strand, which are complementary to each other and typically measure 21–23 base pairs in length. To avoid triggering an immune response, it is essential that siRNAs remain under 30 nucleotides. The double-stranded siRNA interacts with the RNA-induced silencing complex (RISC), prompting the passenger strand to unwind and degrade, while the guide strand binds to the target mRNA, directing its RISC-mediated cleavage. Ultimately, the synthesis of the target protein is hindered [9]. The mechanism of action of siRNAs is shown in Figure 2.

ASOs and siRNAs require modifications to facilitate their uptake by target cells and prevent their degradation by endonucleases. Chemical alterations may modify the pharmacokinetic and pharmacodynamic properties, enhancing their functionality. The structural and chemical differences between ASOs and siRNAs suggest different modification strategies. Nevertheless, backbone modifications, such as phosphorothioate linkages and sugar and base modifications at the 2′ position, remain common. Regarding cellular uptake, N-acetylgalactosamine (GalNAc) is a crucial conjugate that acts as a ligand for the asialoglycoprotein receptor (ASGPR) on hepatocytes, where cholesterol synthesis occurs. Binding to ASGPR allows for rapid internalization of the NAT-GalNAc complex in these cells [7,8,9].

This narrative review delves into the groundbreaking field of RNA-based therapies, highlighting new possibilities for managing dyslipidemias. We aim to discuss the role of the main RNA-based agents that are currently approved or show promise according to preliminary data. The primary molecular targets for these agents are proprotein convertase subtilisin/kexin type 9 (PCSK9), lipoprotein(a) [Lp(a)], apolipoprotein C-III (ApoC-III), and angiopoietin-like protein 3 (ANGPTL3).

## 2. RNA Agents in the Management of Dyslipidemias

An siRNA Therapeutic Targeting PCSK9: *Inclisiran*.

Proprotein convertase subtilisin/kexin type 9 (*PCSK9*) is a serine protease and a member of the subtilisin family. This protein is primarily synthesized in hepatocytes and, to a lesser extent, is present in the small intestine, kidneys, endothelial cells, and vascular smooth muscle cells. It is encoded as a 72 kDa zymogen of 692 amino acids by the *PCSK9* gene, located on the p arm of chromosome 1. Co- and post-translational modifications, including glycosylation in the endoplasmic reticulum and an autocatalytic cleavage, are required for PCSK9 to become functional. Following these modifications, a heterodimer comprising a 62 kDa domain and a non-covalently bound 13 kDa prodomain is formed and secreted from hepatocytes as biologically active PCSK9 [10,11].

PCSK9 contributes to lipid homeostasis by promoting the intracellular degradation of LDL receptors (LDLRs), which are responsible for removing LDL-C from circulation. Gain-of-function and loss-of-function mutations in *PCSK9* demonstrate its impact on the lipid profile and cardiovascular risk. Gain-of-function mutations lead to significantly elevated serum LDL-C levels and an increased risk of cardiovascular disease (CVD), while loss-of-function mutations reduce LDL-C levels and lower the CVD risk [10,11]. Circulating PCSK9 binds to LDLR, and the complex is internalized. For degradation to occur, a recently identified protein called cyclase-associated protein 1 (CAP-1) is essential. Without CAP-1, the PCSK9-LDLR complex is internalized via clathrin-coated vesicles, thus allowing LDLR to be recycled to the cell surface. However, when CAP-1 is bound to PCSK9, a caveolin-mediated pathway initiates LDLR degradation in the endosomes and lysosomes [12].

*Inclisiran* is an siRNA, which inhibits the hepatic synthesis of PCSK9. It was first approved in the EU in December 2020 for adults with primary hypercholesterolemia or mixed dyslipidemia, as an adjunct to diet and statins, for those unable to reach LDL cholesterol targets on maximum tolerated statin doses. It is a long-acting siRNA with a chemically attached trifurcated N-acetylgalactosamine (GalNAc) molecule that acts as a ligand for the ASGPR receptor, facilitating the rapid uptake by hepatocytes. Inclisiran targets the mRNA that translates into PCSK9, thereby hindering its synthesis. With less PCSK9 secreted, more LDL receptors (LDLRs) are available on the cell surface, enhancing the clearance of excess, circulating LDL-C [13].

Phase 2 clinical trials have demonstrated the efficacy, safety, and tolerability of inclisiran, starting with ORION-1 in 2017. ORION-1 is a phase 2, multicenter, double-blind, placebo-controlled study. Inclisiran in six different dosing regimens or a placebo was administered subcutaneously (SC) to 501 patients at high risk of ASCVD. The results showed a dose-dependent reduction in the serum LDL-C levels of up to 52.6%. The maximum reduction was achieved with two-dose 300 mg of inclisiran, at days 1 and 90. In terms of safety, inclisiran and the placebo showed similar rates of adverse events [14]. In the one-year follow-up of the ORION-1 trial (2019), it was demonstrated that a 50% LDL-C reduction was maintained for at least 6 months after two doses of 300 mg of inclisiran on days 1 and 90 [15]. An open-label extension of the ORION-1 trial, ORION-3, conducted in 2023, assessed the effect of long-term inclisiran administration and revealed an average LDL-C reduction of 44.2% over four years, with no serious treatment-related adverse events [16].

Inclisiran’s efficacy was further demonstrated in the phase 3 ORION-10 and ORION-11, ORION-9, ORION-8, and ORION-5 clinical trials, conducted in chronological order.

ORION-10 and ORION-11 were two phase 3, randomized, double-blind, placebo-controlled, parallel group studies conducted in 2020. They were designed to study the effects of the administration of 284 mg of SC inclisiran or a placebo in patients with ASCVD (ORION-10) or an ASCVD equivalent [type 2 diabetes mellitus, familial hypercholesterolemia (FH), or a Framingham Risk Score of ≥20%] and LDL-C of ≥100 mg/dL (ORION-11). Inclisiran was administered on days 1 and 90, and then semiannually for a total of 18 months. By day 510, the LDL-C levels had decreased by 50%. Mild injection-site adverse events were observed more frequently in the inclisiran group compared to the placebo group; 2.6% vs. 0.9% in the ORION-10 trial and 4.7% vs. 0.5% in the ORION-11 trial [17].

ORION-9 was a multicenter, double-blind, placebo-controlled study, which included 482 patients with heterozygous familial hypercholesterolemia (HeFH) and serum LDL-C levels ≥ 100 mg/dL despite treatment with the maximum tolerated dose of a statin, with or without ezetimibe. Patients were randomized to receive 300 mg of subcutaneous (SC) inclisiran or the placebo on days 1, 90, 270, and 450. In the inclisiran group, an LDL-C reduction of 47.9% was observed compared to the placebo group, while serious adverse events did not differ significantly between the two groups [18].

ORION-8 was a phase 3, open-label extension of the ORION-9, ORION-10, ORION-11, and ORION-3 trials. The results revealed that inclisiran led to a 49.4% reduction in LDL-C, with 78.4% of patients achieving their predefined target LDL-C levels. The safety profile was consistent with that of previously conducted studies [19].

ORION-5 was a phase 3, multicenter, randomized, double-blind/open-label study involving 56 patients with homozygous familial hypercholesterolemia (HoFH) and baseline LDL-C > 500 mg/dL before treatment or LDL-C ≥ 130 mg/dL despite treatment with the maximum tolerated statin dose, with or without ezetimibe. Participants were randomized to receive 300 mg of inclisiran or the placebo. By day 150, the PCSK9 levels had decreased by 60.6% compared to the placebo; however, the LDL-C levels were not significantly reduced, apparently due to the limited number of functional LDL receptors in the patients with HoFH [20].

The results of ongoing, phase 3, double-blind, randomized, placebo-controlled outcome trials are eagerly awaited to determine the efficacy of inclisiran in primary and secondary cardiovascular disease prevention.

The VICTORION-1 PREVENT study aims to evaluate the impact of the administration of 300 mg of inclisiran versus a placebo on reducing major adverse cardiovascular events (MACEs) in high-risk patients without established cardiovascular disease. MACEs include cardiovascular death, non-fatal myocardial infarction, non-fatal ischemic stroke, and coronary revascularization. The study aims to recruit 14,000 patients. Its expected completion date is March 2029 [21]. ORION-4 intends to investigate the efficacy of inclisiran in reducing MACEs in approximately 15,000 patients with established CVD, as compared to a placebo. The primary completion date of this study is estimated to be July 2026 [22]. Similarly, the VICTORION-2 PREVENT study aims to assess the role of inclisiran in the secondary prevention of cardiovascular events in 17,000 participants with established CVD compared to a placebo, with an estimated completion date in October 2027 [23].

Pioneering the reduction in lipoprotein(a): pelacarsen, olpasiran, zerlasiran, and lepodisiran.

Lipoprotein(a) [Lp(a)] is a highly atherogenic lipoprotein recognized as an independent causative factor for ASCVD. Structurally similar to LDL-C, Lp(a) consists of a cholesterol ester core encased by apolipoprotein B-100 (ApoB-100) molecules. However, it is distinguished by the addition of apolipoprotein (a) [Apo(a)], which attaches to ApoB-100 via a disulfide bond, imparting Lp(a) with its unique atherogenic and thrombogenic characteristics. Apo(a) is synthesized in hepatocytes and encoded by the *LPA* gene, a highly polymorphic locus that produces variably sized Apo(a) isoforms. Smaller isoforms are linked to elevated Lp(a) levels, leading to hyperlipoproteinemia (a), a condition in which Lp(a) is above 30 mg/dL and is predominantly genetically determined (≈90%) [5]. Here, we will present and discuss the current clinical trial data regarding the efficacy, safety, and tolerability of ASOs and siRNAs targeting Lp(a).

*Pelacarsen*, also known as *AKCEA-Apo(a)-LRx*, is an innovative second-generation ASO designed with a trifurcated N-acetylgalactosamine (GalNAc) molecule for efficient delivery. This GalNAc structure facilitates rapid uptake by asialoglycoprotein receptors (ASGPRs) on hepatocytes, the cells responsible for Lp(a) synthesis. Once inside, pelacarsen effectively reduces the translation of *LPA* messenger RNA into apo(a), thereby targeting Lp(a) production at its source [24,25].

The AKCEA-APO(a)-LRx study investigators conducted a phase 2, double-blind, dose-ranging, placebo-controlled, randomized trial involving 286 patients with established CVD and elevated Lp(a) levels of at least 60 mg/dL. Participants received pelacarsen subcutaneously in five different dosing regimens or a placebo. At six months, pelacarsen achieved a dose-dependent reduction in the Lp(a) levels, ranging from 35% to 80% (*p* < 0.003 to 0.001). Additional key lipid markers also showed significant reductions, with the highest administered dose of pelacarsen (20 mg weekly) oxidized phospholipids on apolipoprotein B (OxPL-apoB) decreased by 88%, oxidized phospholipids on apolipoprotein(a) [OxPL-apo(a)] decreased by 70%, LDL-C levels decreased by 16.4%, and ApoB levels decreased by 10.9% compared to the placebo (all *p* < 0.001). The high-sensitivity C-reactive protein (hsCRP) levels showed no significant difference between the pelacarsen and placebo groups. The pelacarsen and placebo groups also showed no notable differences in their platelet counts, hepatic or renal function, or incidence of influenza-like symptoms. The most common adverse event observed was injection-site reactions [26]. The Lp(a) HORIZON trial is an ongoing, phase 3, multicenter, double-blind, randomized, placebo-controlled study that has enrolled 8323 participants with Lp(a) levels over 70 mg/dL and a history of myocardial infarction, ischemic stroke, or clinically significant symptomatic peripheral artery disease. The trial aims to evaluate the effect of the lowering of pelacarsen-induced Lp(a) on the reduction in major adverse cardiovascular events. Its estimated completion date is 30 May 2025 [27].

Another three innovative agents are being investigated for effective serum Lp(a) reduction, all being siRNAs. *Olpasiran* is a synthetic siRNA molecule that inhibits apo(a) synthesis by blocking the translation of its precursor mRNA, ultimately reducing Lp(a) production within hepatocytes. *Zerlasiran*, previously named SLN360, is a synthetic siRNA that inhibits the translation of the LPA gene transcript, concomitantly reducing Lp(a) production. *Lepodisiran*, which is a 2′-o-me, 2′-fluoro, and unmodified Dicer siRNA, is the latest developed agent targeting Lp(a). All agents are conjugated with a trifurcated GalNAc molecule and have undergone proper structural and chemical modifications for enhanced uptake by hepatocytes [5].

*Olpasiran trials.* The phase 2, randomized, double-blind, placebo-controlled OCEAN(a)-DOSE trial (Olpasiran Trials of Cardiovascular Events and Lipoprotein(a) Reduction—Dose-Finding Study) was designed to assess the efficacy and safety of olpasiran. In this study, 281 patients with established CVD and serum Lp(a) levels > 150 nmol/L were randomly assigned to receive subcutaneous injections of olpasiran in one of four ascending dosage regimens or a placebo. Compared to the placebo, the mean percentage reductions in Lp(a) ranged from 70.5% to 101.1% (*p* < 0.001). Adverse events did not differ significantly between the intervention and placebo groups, with injection-site reactions being the most common [28]. The OCEAN(a)-DOSE outcome trial will assess the effect of olpasiran on the risk of major adverse cardiovascular events (MACEs), including coronary heart disease death, myocardial infarction, and the need for coronary revascularization, compared to the placebo. A total of 7297 eligible patients with established ASCVD and serum Lp(a) levels above 200 nmol/L were enrolled in this phase 3, multicenter, randomized, double-blind, placebo-controlled study. Results are anticipated by December 2026 [29].

*Zerlasiran trials.* APOLLO is a phase 1, single-ascending-dose study enrolling 32 patients with serum Lp(a) levels exceeding 150 nmol/L and no prior history of ASCVD. The study aimed to evaluate the safety and tolerability of zerlasiran. Patients were randomized to receive either zerlasiran or a placebo in five ascending doses, ranging from 30 mg to 600 mg, administered subcutaneously. Zerlasiran led to a dose-dependent Lp(a) reduction of 46% to 96% compared to the placebo, with reductions maintained through day 150. No serious adverse events were observed [30]. The ALPACAR-360 trial, a recently completed phase 2, randomized, placebo-controlled study, which included 178 high-risk ASCVD participants with serum Lp(a) levels ≥ 125 nmol/L, assessed the safety, efficacy, and tolerability of subcutaneously administered zerlasiran. Participants received subcutaneous zerlasiran in doses of either 300 mg every 16 or 24 weeks, or 450 mg every 24 weeks, or a placebo. At 36 weeks, zerlasiran yielded mean Lp(a) reductions of 80% or more across all the dosage regimens, with no serious adverse events reported. The study paves the way for phase 3 trials of zerlasiran [31].

*Lepodisiran trials*. The results of a phase 1, single-ascending-dose, placebo-controlled study were published a year prior to the writing of this manuscript. In this study, 48 healthy participants with Lp(a) levels exceeding 75 nmol/L received subcutaneous injections of lepodisiran (4 mg, 12 mg, 32 mg, 96 mg, 304 mg, or 608 mg) or a placebo. Among participants receiving the highest lepodisiran dose, a mean Lp(a) reduction of 97% was observed and was sustained for nearly a year post-injection. Treatment with lepodisiran appeared to be generally safe [32]. Eagerly awaited ongoing trials include a phase 2, randomized, double-blind, placebo-controlled study enrolling 216 participants with Lp(a) levels above 175 nmol/L to evaluate the efficacy and safety of lepodisiran over 20 months. The results of this study are expected to be posted relatively soon [33]. The latest ongoing study, currently recruiting, is the ACCLAIM-Lp(a) trial, a phase 3, randomized, double-blind, placebo-controlled study aimed at investigating the effect of lepodisiran on reducing major adverse cardiovascular events (MACEs) in 12,500 participants with established ASCVD or high-risk profiles and elevated Lp(a) levels above 175 nmol/L. The study is expected to be completed by March 2031 [34].

Exploring new paths in ApoC-III reduction: *Volanesorsen*, *Olezarsen*, and *Plozasiran*.

Apolipoprotein C-III (ApoC-III) is a glycoprotein encoded by the *APOC3* gene, located on chromosome 11. Primarily synthesized in hepatocytes and intestinal cells, ApoC-III plays a key role in lipid metabolism by inhibiting lipoprotein lipase (LPL) and hepatic lipase (HL). The latter promotes the hydrolysis of VLDL into intermediate-density lipoprotein (IDL) and LDL [35]. LPL is a lipolytic enzyme that catalyzes the breakdown of chylomicrons and very-low-density lipoprotein cholesterol (VLDL-C) into fatty acids. Both quantitative and qualitative defects in LPL are associated with hypertriglyceridemia in the form of familial chylomicronemia [36]. Elevated serum ApoC-III levels have been linked to increased triglycerides, atherosclerosis, and ASCVD, thus making ApoC-III an appealing target for inhibition [35].

*Volanesorsen* is a second-generation ASO. This 20-mer 2′-O-methoxyethyl gapmer has phosphorothioate backbone modifications that enhance its binding to the *APOC3* transcript. This binding blocks translation to ApoC-III through RNAse H-dependent cleavage [37]. Several trials have assessed the efficacy and safety of volanesorsen in patients with familial chylomicronemia syndrome (FCS) or multifactorial chylomicronemia with severe hypertriglyceridemia, which will be discussed further on.

The APPROACH trial was a phase 3, randomized, double-blinded, placebo-controlled study involving 66 patients with FCS. Participants were randomized to receive either a weekly dose of 300 mg of volanesorsen subcutaneously or a placebo. Over three months, the volanesorsen group showed a mean TG reduction of 77%, a mean VLDL-C reduction of 58%, and a mean ApoC-III reduction of 84% (*p* < 0.001, compared to the placebo). However, a mean increase in LDL-C of 136% was observed, presumably due to increased lipolysis. Thrombocytopenia occurred in nearly half of the patients receiving volanesorsen, with the platelet counts falling below 100,000/μL in 45.4% of them, while no such cases were reported in the placebo group. Two patients had platelet counts below 25,000/μL. Injection-site reactions occurred in 60.6% of the patients in the volanesorsen group, compared to none in the placebo group [38]. The COMPASS trial was a phase 3, multicenter, double-blinded, randomized, placebo-controlled trial enrolling 133 patients with multifactorial chylomicronemia and basal TG levels ≥ 500 mg/dL. Volanesorsen in two different dosage regimens or a placebo were administered. From baseline to 3 months, the mean percentage reduction in the TG levels in the volanesorsen group was 71.2%, compared with 0.9% in the placebo group. The ApoC-III levels were reduced by 76.1%, whereas the LDL-C levels were increased by 95.5% due to increased lipolysis (*p* < 0.001, compared to the placebo). Platelet counts below 100,000/μL were observed in 13% of patients receiving volanesorsen, of whom one had a platelet count below 50,000/μL, compared to 5% in the placebo group. Five incidents of acute pancreatitis occurred, all in patients of the placebo group [39]. The long-term efficacy and sustainability of volanesorsen in reducing plasma TG levels was also supported by an open-label extension trial based on the previously mentioned trials [40].

A pooled analysis of four randomized, controlled trials, including the APPROACH and COMPASS trials, and involving a total of 139 patients, demonstrated significant mean percentage reductions in several key parameters compared to the placebo: 74% in the TG levels, 71% in the very-low-density lipoprotein cholesterol (VLDL-C), 80% in the apolipoprotein C-III (ApoC-III) levels, and 69% in the apolipoprotein B48 (ApoB48) levels, an indicator of plasma chylomicron levels. Additionally, a 46% increase in the HDL-C levels was observed. Patients receiving volanesorsen were 13 times more likely to develop thrombocytopenia, highlighting a strong association between volanesorsen and this potentially hazardous adverse event. The most common adverse event reported was injection-site reactions, occurring in 14% of all drug administrations [41]. At the same time as this analysis, the results of the BROADEN study were published. The BROADEN study, a phase 2/3, randomized, placebo-controlled trial involving 40 patients with familial partial lipodystrophy, demonstrated that volanesorsen effectively reduced the TG levels by 88% [42].

Given its demonstrated efficacy, volanesorsen was approved for use in the European Union in May 2019 for treating familial chylomicronemia, hypertriglyceridemia, and familial partial lipodystrophy [43]. However, in 2018, the U.S. Food and Drug Administration (FDA) declined its approval, citing concerns about thrombocytopenia and an increased risk of bleeding [44].

*Olezarsen*, also known as *ISIS 678354* and *AKCEA-APOCIII-LRx*, shares the same nucleic acid sequence as volanesorsen but is conjugated to a trifurcated GalNAc molecule, which enhances its hepatic uptake [45].

Olezarsen has been evaluated in numerous trials. A phase 2, double-blind, randomized, placebo-controlled, dose-ranging study enrolled 114 patients with established or at high risk for CVD and fasting plasma TG levels ranging from 200 to 500 mg/dL. Participants were randomized to receive olezarsen in one of the following doses: 10 mg every 4 weeks, 15 mg every 2 weeks, 10 mg weekly, 50 mg every 4 weeks, or a placebo. At six months, the highest administered dose of olezarsen (50 mg every 4 weeks) resulted in mean percentage reductions of 60% in the plasma TG levels, 74% in the ApoC-III, and 58% in the VLDL-C. The plasma HDL-C levels increased by 30%. No significant differences were observed in the platelet count or liver or renal function between the olezarsen and placebo groups. The most common adverse event was injection-site reactions [45].

The Bridge–TIMI 73a was another phase 2b, double-blinded, randomized, placebo-controlled study that evaluated 154 patients who were either at high risk for CVD with fasting serum TG levels of 200–499 mg/dL or had a TG level ≥ 500 mg/dL. Participants received monthly SC doses of 50 mg or 80 mg of olezarsen, or a placebo. Over six months, olezarsen achieved significant reductions in the lipid parameters: the TG levels decreased by 49.3% and 53.1%, the ApoC-III levels decreased by 64.2% and 73.2%, and the VLDL-C levels decreased by 46.2% and 49.7%, with the 50 mg and 80 mg doses, respectively (all *p* < 0.001 vs. the placebo). TG reductions were evident after one month of treatment and were sustained throughout a 12-month follow-up. No notable changes were observed in the LDL-C levels, platelet counts, or liver and renal function [46].

The Balance study, a phase 3, randomized, double-blinded, placebo-controlled trial, involved 66 patients diagnosed with FCS and with plasma TG levels > 880 mg/dL. Participants received monthly doses of 80 mg or 50 mg of olezarsen or a placebo for 53 weeks. Before the study, 71% of participants had experienced acute pancreatitis over a 10-year period. Six months of olezarsen administration resulted in TG reductions of 43.5% (80 mg, *p* < 0.001) and 22.4% (50 mg, *p* < 0.08), and ApoC-III reductions of 73.7% and 65.5% with the 80 mg and 50 mg olezarsen doses, respectively, compared to the placebo. Patients receiving olezarsen showed a significantly lower rate of acute pancreatitis compared to that of the placebo group, with an 88% reduction in the risk (rate ratio [pooled olezarsen groups vs. placebo], 0.12; 95% CI, 0.02 to 0.66). Four patients in the 80 mg olezarsen group experienced adverse reactions of moderate severity [47].

The accumulated data suggest that olezarsen shows promise as an effective treatment for reducing plasma TG levels. The FDA has granted orphan drug designation to olezarsen for the treatment of FCS [48].

In addition to the completed trials, three ongoing, phase 3, multicenter, randomized, double-blinded, placebo-controlled trials are eagerly awaited. All three aim to evaluate the efficacy of olezarsen in reducing serum TG levels from baseline compared to a placebo. Each trial is listed with its respective clinicaltrials.gov identifier. NCT05552326 and NCT05079919 are expected to be concluded by July 2025. The first trial involves 446 patients, while the second includes 617 patients, all with fasting plasma TG levels ≥ 500 mg/dL. In both studies, the participants are randomized to receive either olezarsen or a placebo for 53 weeks, with a total follow-up of 78 weeks [49,50]. The third trial, NCT05610280, has enrolled 1478 patients with either established or at high risk for CVD and fasting plasma TG levels of 200–500 mg/dL or TG levels ≥ 500 mg/dL. The study consists of a 12-week screening period, a 53-week treatment phase, and a 13-week post-intervention follow-up. It is estimated to be completed by June 2025 [51].

*Plozasiran*, or ARO-APOC3, is another ASO that interferes with the synthesis of ApoC-III at the level of mRNA translation in hepatocytes, which is currently under investigation.

The results of two phase 2b, randomized, double-blinded, placebo-controlled trials are currently available. MUIR, a 48-week trial, studied plozasiran administration in four different dosage regimens (10, 25, and 50 mg at day 1 and at week 12 or 50 mg at day 1 and at week 24) or a placebo in 353 participants with fasting plasma TG levels of 150–499 mg/dL and LDL-C ≥ 70 mg/dL or non-HDL-C ≥ 100 mg/dL. By week 24, plozasiran reduced the TG levels by up to 62.4%, the ApoC-III by up to 78.5%, the non-HDL-C by up to 24.2%, the remnant cholesterol by up to 48.9%, the LDL-C by up to 13.6%, the ApoB by up to 19.1%, and the Lp(a) by up to 23.8%, compared to the placebo. However, the HDL-C increased by up to 45.8% compared to the placebo. The frequency of adverse events did not differ significantly between the two groups [52]. The SHASTA-2 study investigated the administration of plozasiran (10, 25, or 50 mg) or a placebo on day 1 and at week 12 in 226 participants with fasting serum TG levels ≥ 500 mg/dL. By week 24, plozasiran reduced the TG levels by up to 57%, the ApoC-III by up to 77%, the non-HDL-C by up to 20.2%, and the remnant cholesterol by up to 58.8%, as compared to the placebo. Notably, the HDL-C levels were increased by up to 57% compared to the placebo. Plozasiran was generally well tolerated throughout the study [53].

The PALISADE study is an ongoing, phase 3, multicenter, double-blinded, randomized, placebo-controlled trial that has recruited 75 patients with FCS and a mean fasting TG level of 2044 mg/dL. The study aims to evaluate the efficacy and safety of plozasiran in patients with FCS. Participants are randomized to receive plozasiran (25 mg or 50 mg) or a placebo every three months. Patients completing the randomization period may be eligible to enroll in a two-year, open-label extension trial, during which only plozasiran will be administered. By month 10, plozasiran achieved a median TG reduction by up to 80% and a mean ApoC-III reduction by up to 96%. Furthermore, plozasiran significantly reduced the odds of acute pancreatitis compared to the placebo, with an 83% reduction in the risk (OR: 0.17; *p* = 0.03) [54,55].

siRNA-based inhibition of ANGPTL3: *Zodasiran* and *Solbinsiran*.

Angiopoietin-like proteins (ANGPTLs) are a family of secreted glycoproteins (ANGPTL1-8), structurally similar to angiopoietins, which play a key role in angiogenesis. The ANGPTL3-4-8 subgroup, characterized by high sequence homology, regulates lipid metabolism by inhibiting lipoprotein lipase (LPL) and endothelial lipase (EL) in various tissues. LPL, located on the luminal side of the vascular endothelium, and EL, active on the endothelial surface, hydrolyze triglycerides in chylomicrons and very-low-density lipoproteins (VLDLs). In addition, EL also hydrolyzes phospholipids. ANGPTL3, a 70 kDa glycoprotein expressed in the liver during both embryonic development and adulthood, requires binding with ANGPTL8 to enhance its function, though it can also independently inhibit EL. Loss-of-function variants in ANGPTL3, identified in large-scale studies, are associated with familial combined hypolipidemia (FCH) and reduced coronary artery disease (CAD) risk; however, no link has been established between gain-of-function mutations in ANGPTL3 and dyslipidemia, such as familial combined hyperlipidemia (FCHL) [56].

*Zodasiran*, or ARO-ANG3, an siRNA targeting ANGPTL3 synthesis, is currently under evaluation. The ARCHES-2 study, a phase 2, dose-ranging, double-blinded, randomized, placebo-controlled trial, assessed the safety and efficacy of zodasiran in 204 adults with mixed hyperlipidemia. Participants were randomized to receive subcutaneous zodasiran (50, 100, or 200 mg) or a placebo on day 1 and week 12, with follow-up through week 36. At week 24, zodasiran demonstrated significant mean percentage reductions across several lipid parameters: up to 63.1% in the TG levels, up to 73.7% in ANGPTL3, up to 36.4% in non-HDL-C, up to 19.9% in LDL-C, and up to 20% in Lp(a). Importantly, no increase in hepatic fat was observed, although a transient elevation in the glycated hemoglobin (HbA1C) levels was noted. Zodasiran was generally well tolerated throughout the study [57]. An ongoing, phase 2, open-label study, the Gateway study, has recruited 18 patients with HoFH and LDL-C > 100 mg/dL to receive zodasiran for 36 weeks; after completing the first 36-week treatment phase, participants may choose to enter a 24-month extension phase, during which they will receive up to eight open-label doses of zodasiran. The study aims to evaluate the efficacy and safety of zodasiran in patients with HoFH, and its expected completion date is May 2025 [58].

*Solbinsiran* or *LY3561774* is an siRNA conjugated with GalNAc, which targets ANGPTL3 and is under investigation [59]. A phase 1, multicenter, double-blind, randomized, placebo-controlled study enrolled 40 individuals with fasting triglyceride levels of 150–499 mg/dL and LDL-C ≥ 70 mg/dL. Participants were randomized to receive solbinsiran in one of seven dose regimens or a placebo. The study observed dose-dependent reductions in the ANGPTL3 by up to 86%, in the TG levels by up to 73%, in the non-HDL-C by up to 46%, and in the ApoB by up to 36%. These reductions were sustained for 169 days, maintaining a dose-dependent effect throughout the study period. Adverse events were reported in 17–67% of participants in the solbinsiran group and in 20–50% in the placebo group, with most events being mild in severity [60]. A recently completed phase 2b, multicenter, double-blinded, placebo-controlled, parallel-group study, the PROLONG-ANG3 study, administered three different dosage regimens of solbinsiran or a placebo to 175 patients with mixed dyslipidemia to assess the efficacy and safety of the agent. The results of this study are expected to be posted relatively soon [61].

A summary of the novel RNA-based therapies in the management of dyslipidemias is shown in Table 1.

A summary of the key clinical trials of the novel RNA-based therapies discussed in this review—in order of reference—is shown in Table 2.

## 3. Conclusions

RNA-based therapies for dyslipidemias, including ASOs and siRNAs, represent a revolutionary advancement in the treatment of lipid disorders. These cutting-edge therapies primarily target four crucial components involved in lipid metabolism: PCSK9, Lp(a), ApoC-III, and ANGPTL3. The results emerging from clinical trials evaluating these novel RNA therapies have been overwhelmingly positive, fueling optimism for their future integration into routine clinical practice in the near future. As these therapies demonstrate promising efficacy, they are expected to significantly improve the management of dyslipidemias and address gaps in the current treatment strategies. Moreover, ongoing clinical outcome studies hold great potential for further elucidating the role of RNA-based treatments in reducing cardiovascular risk, a critical concern for patients with lipid abnormalities. With their ability to precisely target key pathways involved in lipid regulation, these therapies offer the possibility of more personalized, effective treatment approaches. As research progresses, RNA-based therapies are set to revolutionize the landscape of lipid management, ultimately improving long-term outcomes and enhancing the quality of life for patients worldwide.

In terms of future research directions, there are several key areas for investigation. Evaluation of the long-term safety, effectiveness, and interaction of RNA-based therapies with other chronic conditions and treatments, as in the case of diabetes and hypertension, is necessary. As RNA therapies move closer to clinical use, it is important to consider their long-term effects, possible misuse, and unintended risks. Clear rules and guidelines are needed to ensure they are used safely and responsibly. Another key focus should be exploring how RNA-based therapies work alongside traditional treatments like statins, fibrates, or PCSK9 inhibitors to obtain the best results. Personalizing these therapies is another exciting but challenging area. By identifying genetic factors that affect how people respond to treatment, we can create more tailored and effective plans. Developing tools like algorithms or biomarkers to predict individual responses will be crucial. Finally, the high cost of RNA-based therapies is a major challenge, especially in developing countries. Researchers should work on finding ways to make these treatments more affordable and easier to produce so they can be more widely available without compromising their effectiveness and safety.

## Figures and Tables

**Table 1:** Novel RNA-based therapies in the management of dyslipidemias.

Type of Nucleic Acid Therapeutic	Agent	Target
Antisense Oligonucleotides (ASOs)	Pelacarsen (AKCEA-APO(a)-LRx, APO(a)-LRx, TQJ230)	Lp(a)
	Volanesorsen	ApoC-III
	Olezarsen (ISIS 678354, AKCEA-APOCIII-LRx)	
	Plozasiran (ARO-APOC3)	
Small Interfering RNAs (siRNAs)	Inclisiran	PCSK9
	Olpasiran	Lp(a)
	Zerlasiran (SLN360)	
	Lepodisiran (LY3819469)	
	Zodasiran (ARO-ANG3)	ANGPTL3
	Solbinsiran (LY3561774)	

**Table 2:** Clinical trials—novel RNA-based therapies in the management of dyslipidemias.

Target/Name	Study Design	Purpose/Intervention	Results
PCSK9			
Inclisiran			
[14] ORION-1	Phase 2, multicenter, double-blinded, placebo-controlled (501 patients at high risk for CVD)	Inclisiran (SC administration, 300 mg) vs. placebo	Dose-dependent LDL-C reduction up to 52.6%; similar safety profiles.
[15] Ray et al.: One-Year Follow-up of the ORION-1 Randomized Clinical Trial	Phase 2, multicenter, double-blinded, placebo-controlled	Inclisiran (300 mg subcutaneous injection, days 1 and 90) vs. placebo	50% LDL-C reduction at 6 months, 30% at 12 months; optimal dosing regimen identified.
[16] ORION-3	Open-label extension of ORION-1	Inclisiran (long-term administration)	44.2% LDL-C reduction over 4 years; no serious treatment-related AEs.
[17] ORION-10 & ORION-11	Phase 3, randomized, double-blind, placebo-controlled in patients with established CVD or an ASCVD equivalent	Inclisiran (284 mg, SC injections at day 1, 90, and every 6 months) vs. placebo	50% LDL-C reduction; mild injection-site AEs in inclisiran group (ORION-10: 2.6% vs. 0.9%—ORION-11: 4.7% vs. 0.5%).
[18] ORION-9	Phase 3, multicenter, double-blinded, placebo-controlled (482 patients with HeFH and LDL-C > 100 mg/dL)	Inclisiran (300 mg, multiple doses) vs. placebo	47.9% LDL-C reduction; similar, non-significant serious AEs between groups.
[19] ORION-8	Phase 3, open-label extension of ORION-9, 10, 11	Inclisiran (300 mg) vs. placebo	49.4% LDL-C reduction; 78.4% of patients achieved LDL-C targets; safety data consistent with previous studies.
[20] ORION-5	Phase 3, randomized, double-blind/open-label study (56 patients with HoFH and baseline LDL-C > 500 mg/dL before treatment or LDL-C ≥ 130 mg/dL despite treatment with the maximum tolerated statin dose, with or without ezetimibe)	Inclisiran (300 mg) vs. placebo	60.6% reduction in PCSK9, but no significant LDL-C reduction.
[21] VICTORION-1 PREVENT	Phase 3, randomized, double-blind, placebo-controlled (estimated recruitment: 14,000 patients)	Inclisiran (300 mg) vs. placebo	Ongoing. Aims to evaluate the effect of inclisiran on MACE reduction in high-risk patients without CVD. Estimated completion in 2029.
[22] ORION-4	Phase 3, randomized, double-blind, placebo-controlled (estimated recruitment: 15,000 patients)	Inclisiran (300 mg) vs. placebo	Ongoing. Aims to evaluate the effect of inclisiran on MACE reduction in established CVD patients. Estimated completion in 2049.
[23] VICTORION-2 PREVENT	Phase 3, randomized, double-blind, placebo-controlled (estimated recruitment: 17,000 patients)	Inclisiran (300 mg) vs. placebo	Ongoing. Secondary prevention of cardiovascular events in established CVD patients. Estimated completion in 2027.
Lipoprotein (a)			
Pelacarsen			
[26] AKCEA-APO(a)-LRx Study	Phase 2, double-blind, dose-ranging, placebo-controlled, randomized trial (286 patients with established CVD and Lp(a) > 60 mg/dL)	Pelacarsen (20 mg weekly or other dosing regimens), subcutaneously vs. placebo	Dose-dependent reduction in Lp(a) by 35% to 80% (*p* < 0.003 to 0.001), LDL-C reduced by 16.4%, ApoB reduced by 10.9%, OxPL-apoB reduced by 88%, OxPL-apo(a) reduced by 70%. No significant difference in hsCRP or major adverse events. Common AE: injection-site reactions.
[27] Lp(a) HORIZON Trial	Ongoing, phase 3, multicenter, double-blind, randomized, placebo-controlled (8323 patients)	Pelacarsen vs. placebo, subcutaneously	Ongoing. Aims to evaluate the effect of pelacarsen on MACEs in participants with ASCVD and Lp(a) > 70 mg/dL. Estimated completion date: May 30, 2025.
Olpasiran			
[28] OCEAN(a)-DOSE Trial	Phase 2, randomized, double-blinded, placebo-controlled (281 patients)	Olpasiran in four ascending subcutaneous dosage regimens vs. placebo	Lp(a) reduction ranged from 70.5% to 101.1% (*p* < 0.001). No significant difference in adverse events; most common: injection-site reactions.
[29] OCEAN(a)-DOSE Outcomes Trial	Ongoing, phase 3, multicenter, randomized, double-blinded, placebo-controlled (7297 patients)	Olpasiran vs. placebo, subcutaneously	Ongoing. Aims to evaluate the impact of olpasiran on MACE. Results expected by December 2026.
Zerlasiran			
[30] APOLLO Study	Phase 1, single-ascending-dose (32 patients)	Subcutaneous zerlasiran administration in five ascending doses (30–600 mg) vs. placebo	Dose-dependent Lp(a) reduction of 46% to 96%, maintained through day 150. No serious adverse events observed.
[31] ALPACAR-360	Phase 2, randomized, placebo-controlled (178 patients)	Subcutaneous zerlasiran administration: 300 mg every 16 or 24 weeks, or 450 mg every 24 weeks, vs. placebo	At 36 weeks, Lp(a) reductions of 80% or more across all regimens. No serious adverse events reported.
Lepodisiran			
[32] Nissen et al.	Phase 1, single-ascending-dose, placebo-controlled (48 healthy participants)	Subcutaneous lepodisiran (4–608 mg) vs. placebo	Mean Lp(a) reduction of 97% at the highest dose, sustained for nearly a year. Treatment was generally safe.
[33] ClinicalTrials.gov ID: NCT05565742	Phase 2, randomized, double-blind, placebo-controlled (216 participants with Lp(a) ≥ 175 nmol/L)	Subcutaneous lepodisiran over 20 months vs. placebo	Results pending.
[34] ACCLAIM-Lp(a) Trial	Ongoing, phase 3, randomized, double-blind, placebo-controlled (12,500 participants with established CVD or at high CVD risk and Lp(a) ≥ 175 nmol/L)	Subcutaneous lepodisiran vs. placebo to assess impact on major adverse cardiovascular events (MACEs)	Ongoing. Aims to evaluate the effect of lepodisiran on MACE reduction. Completion expected by March 2031.
ApoC-III			
Volanesorsen			
[38] APPROACH Trial	Phase 3, randomized, double-blind, placebo-controlled (66 patients with FCS)	Subcutaneous volanesorsen (300 mg weekly) vs. placebo	77% TG reduction, 58% VLDL-C reduction, 84% ApoC-III reduction (*p* < 0.001). LDL-C increased by 136%. Thrombocytopenia in 45.4%; injection-site reactions in 60.6%.
[39] COMPASS Trial	Phase 3, multicenter, double-blind, randomized, placebo-controlled (133 patients with multifactorial chylomicronemia)	Subcutaneous volanesorsen vs. placebo	71.2% TG reduction, 76.1% ApoC-III reduction, 95.5% LDL-C increase (*p* < 0.001). Thrombocytopenia in 13%. No cases of pancreatitis in volanesorsen group.
[40] Witzum et al.	Based on phase 3 trials (number of patients not specified)	Subcutaneous volanesorsen	Sustained reduction in TG levels over long-term use.
[41] Calcaterra et al.	Analysis of four randomized trials (139 patients)	Subcutaneous volanesorsen vs. placebo	TG reduced by 74%, VLDL-C by 71%, ApoC-III by 80%, ApoB48 by 69%; HDL-C increased by 46%. Thrombocytopenia strongly associated with volanesorsen use.
[42] BROADEN Study	Phase 2/3, randomized, placebo-controlled (40 patients with familial partial lipodystrophy)	Subcutaneous volanesorsen	TG reduction by 88%.
Olezarsen			
[45] Tardif et al.	Phase 2, double-blind, randomized, placebo-controlled (114 patients with established/high-risk CVD; TG, 200–500 mg/dL)	Olezarsen: 10 mg monthly, 15 mg every two weeks, 10 mg weekly, 50 mg monthly, or placebo	At 6 months: 60% TG reduction, 74% ApoC-III reduction, 58% VLDL-C reduction. HDL-C increased by 30%. No differences in platelet counts, liver, or renal function. Injection-site reactions were the most common adverse event.
[46] Bridge–TIMI 73a	Phase 2b, double-blind, randomized, placebo-controlled (154 patients with high-risk CVD; TG, 200–499 mg/dL or ≥500 mg/dL)	Olezarsen: SC doses of 50 mg or 80 mg vs. placebo	At 6 months: TG reduced by up to and 53.1%, ApoC-III reduced by up to 73.2%, and VLDL-C reduced by up to 49.7% (*p* < 0.001 vs. placebo). Lipid reductions were sustained for 12 months. No notable safety concerns.
[47] Balance	Phase 3, randomized, double-blind, placebo-controlled (66 patients with FCS; TG > 880 mg/dL)	Olezarsen: monthly doses of 80 mg or 50 mg vs. placebo	At 6 months: TG reduced by 43.5% (80 mg, *p* < 0.001) and 22.4% (50 mg, *p* < 0.08); ApoC-III reduced by 73.7% (80 mg) and 65.5% (50 mg). 88% reduction in acute pancreatitis risk. Moderate adverse events in four patients (80 mg group).
[49] ClinicalTrials.gov ID: NCT05552326	Phase 3, randomized, double-blind, placebo-controlled (446 patients; fasting plasma TG ≥ 500 mg/dL)	Olezarsen vs. placebo; 53-week treatment phase with 78 weeks total follow-up.	Ongoing. Aims to evaluate efficacy of olezarsen (vs. placebo) in reducing serum TG levels from baseline. Estimated completion: July 2025.
[50] ClinicalTrials.gov ID: NCT05079919	Phase 3, randomized, double-blind, placebo-controlled (617 patients; fasting plasma TG ≥ 500 mg/dL)	Olezarsen vs. placebo; 53 week-treatment with a 78-week total follow-up.	Ongoing. Aims to evaluate efficacy of olezarsen (vs. placebo) in reducing serum TG levels from baseline. Estimated completion: July 2025.
[51] ClinicalTrials.gov ID: NCT05610280	Phase 3, randomized, double-blind, placebo-controlled (1478 patients with either established CVD or of high CVD risk and TG 200–500 mg/dL or with TG ≥ 500 mg/dL)	Olezarsen vs. placebo; 53-week treatment phase and 13-week post-intervention follow-up	Ongoing. Aims to evaluate efficacy of olezarsen (vs. placebo) in reducing serum TG levels from baseline. Estimated completion: June 2025.
Plozasiran			
[52] MUIR	Phase 2b, randomized, double-blind, placebo-controlled (353 patients; fasting plasma TG, 150–499 mg/dL, LDL-C ≥ 70 mg/dL, or non-HDL-C ≥ 100 mg/dL)	Plozasiran (10, 25, 50 mg at day 1 and week 12; or 50 mg at day 1 and week 24) vs. placebo	TG reduced by 62.4%, ApoC-III by 78.5%, non-HDL-C by 24.2%, and LDL-C by 13.6% at 24 weeks. Adverse events similar between groups.
[53] SHASTA-2	Phase 2b, randomized, double-blind, placebo-controlled (226 patients; fasting serum TG ≥ 500 mg/dL)	Plozasiran (10, 25, or 50 mg on day 1 and week 12) vs. placebo	Dose-dependent reductions: TG by up to 57%, ApoC-III by up to 77%, non-HDL-C by up to 20.2%. HDL-C increased by up to 57%. Well tolerated.
[54,55] ClinicalTrials.gov ID: NCT05089084—PALISADE	Phase 3, multicenter, double-blind, randomized, placebo-controlled (75 patients; mean fasting TG = 2044 mg/dL; familial chylomicronemia syndrome)	Plozasiran (25 mg or 50 mg every 3 months) vs. placebo	By month 10: TG reduced by up to 80%, ApoC-III by up to 96%, and acute pancreatitis risk by 83% (OR 0.17, *p* = 0.03). Open-label, extension, ongoing.
ANGPTL3			
Zodasiran			
[57] ARCHES-2	Phase 2, dose-ranging, double-blind, randomized, placebo-controlled (204 patients; mixed hyperlipidemia)	Zodasiran (50, 100, or 200 mg SC on day 1 and week 12) vs. placebo	At week 24: TG reduced by 63%, ANGPTL3 by 73.7%, non-HDL-C by 36.4%, LDL-C by 20%, and Lp(a) by 20%. No hepatic fat increase; transient HbA1C elevation. Well tolerated.
[58] Gateway	Phase 2, open-label (18 patients; homozygous familial hypercholesterolemia (HoFH), LDL-C > 100 mg/dL)	Zodasiran (SC administration for 36 weeks, followed by up to eight open-label doses in a 24-month extension)	Ongoing. Aims to evaluate the efficacy and safety of zodasiran in HoFH. Estimated completion: May 2025.
Solbinsiran			
[60] Ray, Ruotolo et al.	Phase 1, multicenter, double-blind, randomized, placebo-controlled (40 individuals; fasting TG 150–499 mg/dL; LDL-C ≥ 70 mg/dL)	Solbinsiran (seven dose regimens) vs. placebo	Dose-dependent reductions: ANGPTL3 by up to 86%, TG by up to 73%, non-HDL-C by up to 46%, ApoB by up to 36%. Sustained for 169 days. Most adverse events mild, reported in 17–67% (solbinsiran) vs. 20–50% (placebo).
[61] Clinicaltrials.gov ID: NCT05256654—PROLONG-ANG3	Phase 2b, multicenter, double-blind, placebo-controlled, parallel-group (175 patients; mixed dyslipidemia)	Solbinsiran (three dosage regimens) vs. placebo	Study completed. Publication of results pending.
